# Supplementary material for: Identification of a pre-active conformation of a pentameric channel receptor
Source: eLife. 2017 Mar 15;6:e23955. doi: 10.7554/eLife.23955 (PMC5398890; doi:10.7554/eLife.23955)
Supplement: Supplementary file 1. — DOI: http://dx.doi.org/10.7554/eLife.23955.025 [file elife-23955-supp1.docx]

|  | **Bimane-135** |
| --- | --- |
| **Data collection** |  |
| Resolution range (Å) | 12 - 2.6 (2.692 - 2.6) |
| Space group | C 1 2 1 |
| Unit cell | a = 182.034; b = 134.075; c = 159.945; β = 102.51 |
| Total reflections | 224126 (22382) |
| Unique reflections | 113718 (11338) |
| Multiplicity | 2.0 (2.0) |
| Completeness | 0.98 (1.00) |
| Mean I/σ(I) | 13.04 (1.02) |
| Wilson B-factor | 69.01 |
| R-merge | 0.03618 (0.636) |
| R-meas | 0.05117 (0.8994) |
| CC1/2 | 0.999 (0.836) |
| CC* | 1 (0.954) |
| **Refinement** |  |
| R-work | 0.204 |
| R-free | 0.236 |
| CC(work) | 0.963 |
| CC(free) | 0.958 |
| Number of non-hydrogen atoms | 13647 |
| macromolecules | 12656 |
| ligands | 846 |
| Protein residues | 1555 |
| **Geometry**  RMS (bonds) (Å) | 0.01 |
| RMS (angles) (°) | 1.14 |
| Ramachandran favored (%) | 96 |
| Ramachandran allowed (%) | 3.9 |
| Ramachandran outliers (%) | 0.065 |
| Molprobity score | 99th percentile |
| **Average B-factor** | **87.88** |
| macromolecules | 85.58 |
| ligands | 124.07 |
| solvent | 77.83 |

**Supplementary file 1A:** X-ray data collection and refinement statistics**.**

| **Mutant** | **d_bimane_-d_trp_ pH 7 (Å)** | **F/F_no quencher_ pH 7** | **n** | **d_bimane_-d_trp_ pH 4 (Å)** | **F/F_no quencher_ pH 4** | **n** |
| --- | --- | --- | --- | --- | --- | --- |
| R133C S44W | 14 | 1.01 ± 0.03 | 3 | 12 | 1.03 ± 0.04 | 3 |
| R133C Y23W | 15 | 0.95 ± 0.05 | 3 | 12 | 0.86 ± 0.04 | 3 |
| R133C Q101W | 13.5 | 0.95 ± 0.04 | 3 | 13 | 0.79 ± 0.04 | 3 |
| R133C L103W | 9.5 | 0.65 ± 0.02 | 4 | 8.5 | 0.31 ± 0.03 | 4 |
| V135C W72 | 10 | 0.66 ± 0.03 | 5 | 10 | 0.40 ± 0.06 | 5 |
| D136C S93W | 9 | 0.34 ± 0.09 | 4 | 6 | 0.2 ± 0.1 | 4 |
| D136C Q101W | 13 | 0.81 ± 0.02 | 4 | 9.5 | 0.30 ± 0.04 | 4 |
| D136C D178W | 14 | 0.54 ± 0.01 | 3 | 10.5 | 0.52 ± 0.01 | 3 |
| K33C W160 | 17 | 1.02 ± 0.02 | 4 | 14.5 | 0.42 ± 0.03 | 4 |
| E243C F238W | 12 | 0.462 ± 0.007 | 3 | 11.5 | 0.662 ± 0.003 | 3 |
| E243C L241W | 7 | 0.13 ± 0.01 | 3 | 9 | 0.26 ± 0.03 | 3 |
| P250C Y197 | 8 | 0.60 ± 0.05 | 4 | 11 | 0.93 ± 0.04 | 4 |

**Supplementary file 1B:** Variations of distances and fluorescence between pH 7 and pH 4. The distances were measured between Cβ of the Bimane-Cys position and the inserted/endogenous quencher, using only subunits A and B of the GLIC_pH 7_ and GLIC_pH 4_ structures. The data are presented only for the functional mutants. n represents the number of experiments. The fluorescence mean values are calculated as (F with quencher)/(F without quencher), error values are calculated as standard deviations.
